# Wolf contact in horses at permanent pasture in Germany

**DOI:** 10.1371/journal.pone.0289767

**Published:** 2023-08-10

**Authors:** Konstanze Krueger, Theo Gruentjens, Enno Hempel

**Affiliations:** 1 Department of Equine Economics, Faculty of Agriculture, Economics and Management, Nuertingen-Geislingen University, Nürtingen, Germany; 2 Association for the Promotion of Research on Horses and Wolves (VFWPW), Former AK Pferd & Wolf Until 2020, Verden, Lower Saxony, Germany; Universidade Federal de Mato Grosso do Sul, BRAZIL

## Abstract

Wolves returned to Germany in 2000, leading to fear in German horse owners that their horses could be in danger of wolf attacks or panic-like escapes from pastures when sighting wolves. However, reports from southern European countries indicate that wolf predation on horses diminishes with increasing presence of wildlife. Therefore, we conducted a long-term, filed observation between January 2015 and July 2022 on 13 non breeding riding horses, mares and geldings, kept permanently on two pastures within the range of wildlife and a stable wolf pack with annual offspring. Wildlife cameras at the fences of the pastures made 984 times recordings of wolves and 3151 times recordings of wildlife in and around the pastures. Between 1 January 2022 and 23 March 2022 we observed two stable horse groups. Pasture 1 was grazed by five horses of mixed breed, four mares and one gelding, with the median age of 8 years (min. = 6y, max. = 29y). Pasture 2 was grazed by eight heavy warmbloods and draught horses, three mares and five geldings, with the median age of 16 years (min. = 13y, max. = 22y). During this period no wolf was recorded at pasture 2, but wild boar several times, whereas at pasture 1, wolves were recorded 89 times, and for the wildlife mostly hare. Wolves may have avoided pasture 2 because of the presence of wild boar or because the large group of older, heavy breed horses may have formed a stable, protective group. The latter needs to be confirmed in a follow-up field observation, which records anti-predator behavior and welfare indicators in horses. In conclusion, wolves did not attack the mature horses on pastures with plenty of wildlife and the horses did not respond to the presence of wolves with visible signs of reduced welfare or panic. This indicates that wolves may prefer to prey on easily accessible wildlife around and at horse pastures and that Central European horses become accustom to the presence of non-hunting wolves.

## Introduction

Wolves returned to Germany in 2000 [1] causing worries in German horse owners. Horse owners feared, and still fear, that horses in Germany might be in danger of wolf attacks or could cause serious car accidents on local roads or even highways [2–5] at panic-like escapes from their pastures when sighting wolves [2, 3].

Indeed, wolves were reported to prey on horses [6–11]. Even though horses are generally not considered to be the wolves’ primary prey species, in some areas of southern Europe horses can constitute up to 93% of their diet [7, 9]. Farmers in northern Spain and Portugal reported losses of 59% of their foals to wolves [12], with 41% of the carcasses being found and assigned to wolf predation [13]. In Spain and Portugal, wolves preyed mostly on foals and juveniles of Barrenos, which are local breeds of rather small horses that roam freely throughout the year without any predator protection measures [8]. Wolf packs may even specialize on hunting horses in these areas [3, 7, 9, 14] and may learn how to attack mature horses [8, 9, 13].

In military areas of North East Germany, single wolves and pairs of wolves started to established stable habitats in 2002 [15]. From there, wolves spread out all over Germany, moving into new habitats. By the monitoring year 2020–2021 the German wolf population had increased to 158 packs (with 5 animals on average = the alpha pair and 3 juveniles), 27 pairs, and 20 territorial individuals [16]. In Germany, the first attacks on horses by wolves were reported in 2016 and the frequency of attacks increased annually [3, 16]. In the monitoring year 2020–2021, 18 wolf attacks were documented, with 11 genetically confirmed cases [16]. The confirmed cases were eight Shetland ponies (7 died, 1 was injured), two Konik foals (1 died, 1 was injured), one 20 year old pony-mix (died), and a 40 year old small horse (injured) [16].

Increasing population sizes of wildlife in southern Europe were suggested to diminish predation pressure on horses, especially in areas of Italy, Spain and Portugal where farm animals comprise the majority of wolf diet [9, 11, 13, 14, 17]. In fact, wolves preferentially preyed on roe deer, red deer, wild boar, fallow deer and mouflon in Central Europe, where farm animals comprised only 2% of the wolf diet (monitored for Germany in 2020–2021 [16]). In the German county Bautzen, prey animals for wolves were reported to comprise mostly roe deer, red deer, and wild boar [18, 19].

Whether horses can protect themselves efficiently from wolf attacks might depend on the structure of horse groups [3, 13]. Horses form herds of between 40 and up to 1000 animals [20–22]. In a natural setting, the herds are structured in subgroups, which could be either harem groups, with 1–5 males, several mares and their offspring, or bachelor groups composed of males only [20–22]. Under human management regimes, single-sex groups as well as mixed groups of mares and, geldings without breeding animals, such as stallions and breeding mares, are common. Stallion groups on pasture are rare [23–25]. The numbers of horses kept on pasture increase continuously in Germany [26].

It remained questionable whether horses of central Europe, which lack experience with predation, would show anti-predator behaviour or whether they would rather flee in panic at first sight or scent of a wolf [2]. However, pilot studies on horse anti-predator reactions revealed comparable reactions in Central European horses to Southern European horses [27–30]. At wildlife presence, a group of mares with foals were shown to enhance group formation by reducing individual distances and movement speed [28, 31]. To the presentation of wolf urine [27] and wolf howl [29], horses responded with only remote increases in heart rate [27, 29] and glucocorticoid rates as well as decreases in heart rate variability [29], which indicates that they experienced fear but not panic [27, 29]. Furthermore, anti-predator responses differed between horse breeds [29, 30]. Generally, the strength of horse reactions and attentiveness to predator cues increased with the percentage of thoroughbred ancestry [30].

For gaining more insight into the reaction of Central European riding horse groups to wildlife and wolf presence, we continuously recorded non breeding mares and geldings of mixed breeds and ages with wildlife cameras, which were permanently kept on pastures within the range of a stable wolf pack with annual offspring between 2015 and 2022 [28, 31]. Along with the horses, wildlife incidences at and around the pastures were documented. Six wildlife cameras of the type Wildblick and Secacam raptor were placed around two horse pastures. Therefore, we raised the questions: a) Can we record wolves outside or inside of horse pastures? b) How would horses react to wolf presence or even wolf attack? c) Would wolves frequent horse pastures differently when wildlife differs at the pastures? d) Would wolves attack horses in areas with plenty of wildlife e) Do wolves incidences on horse pastures differ when horses differ in breed, shape and/or age?

## Material and method

### Research area

**Fig 1:**
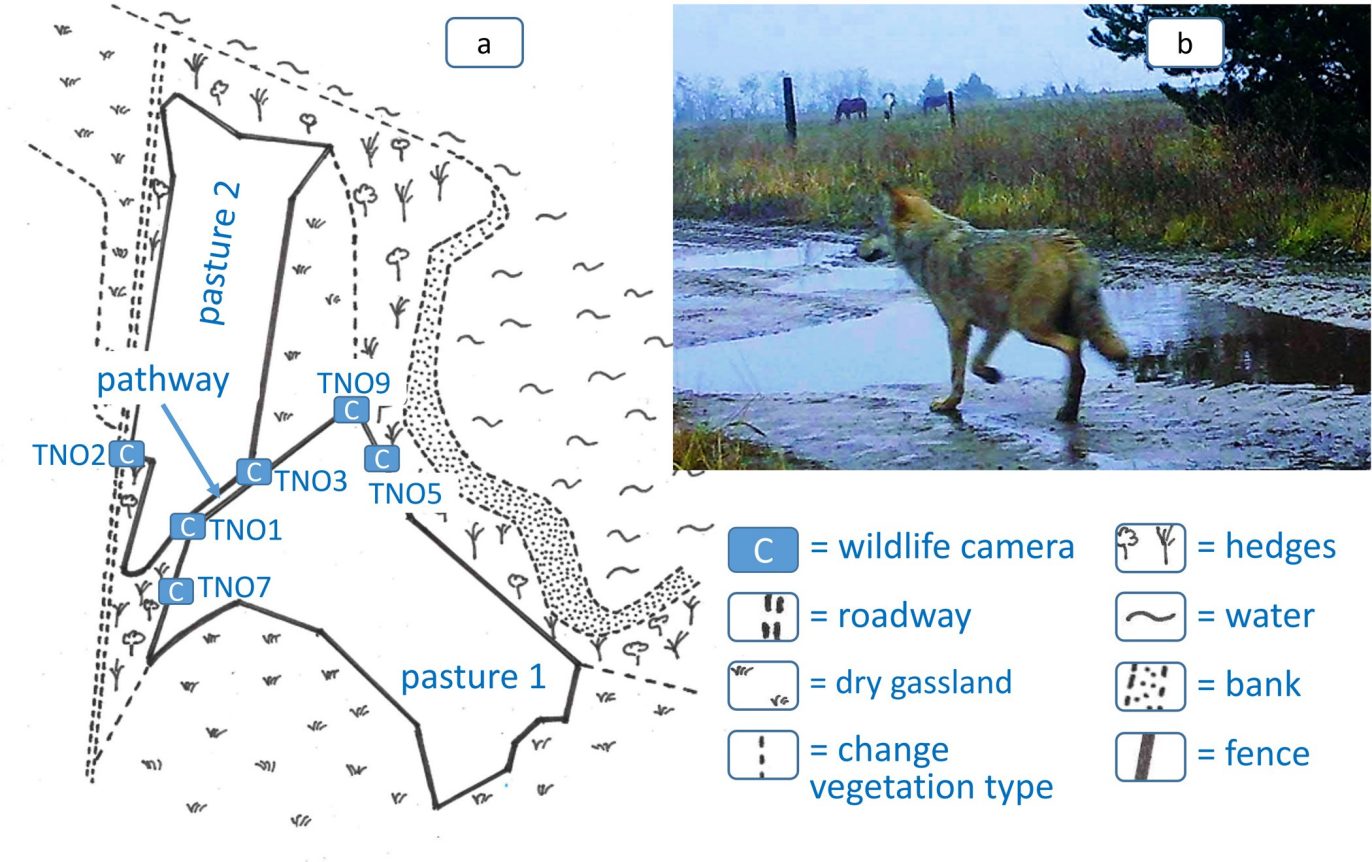
Observation area. a) shows a drawing of the observation area with the cameras TNO5, TNO7 and TNO9 at pasture 1, the camera TNO2 at pasture 2 and the cameras TNO1 and TNO3 at the pathway between the pastures. b) shows a wolf passing pasture 2 on the roadway, recorded by wildlife camera TNO2. Grazing horses are visible at the upper half of the picture (see more wolf and horse recordings at S1 File).

Between January 2015 and July 2022 field observations and camera recordings of horses and wildlife were conducted in the east of Germany, between Dresden and Cottbus. The horses were kept on two pastures: pasture 1 with the size of 19.78 ha and pasture 2 with a size of 15.23 ha (34.91 ha in total). The two pastures were separated by a 265 m long pathway. The vegetation of the pastures was classified as dry grassland and the pastures were surrounded by dry grassland, occasional hedges and a lake in the east (Fig 1).

The pastures were conventionally fenced with wire. The fence of pasture 1 was comprised of three electrified wires at 40, 70, and 120 cm above ground, whereas the fence of pasture 2 had four electrified wires at 40, 70, 100, 130 cm above ground (Fig 1 and S1 File). The fences cannot be considered wolf deterrent fencing, because wolves can easily crawl underneath the lowest electrified wire. Wolf deterrent fencing would need to fix the lowest electric wire at a height of not more than 20 cm above ground [32].

#### Animals

On average 13 horses of varying breeds, age and sex were kept permanently on the two pastures and grazed the natural vegetation. They were provided with hay, water and mineral-licking stones ad libitum. Annual veterinary checks proved all horses to be in good health. During the comparative observation period between 1 January 2022 and 23 March 2022, the composition of the horse groups did not change. The horses numbered 13 animals and were comprised of one pony, two warmbloods, three heavy warmbloods and six draught horses (for one, the breed and age was unknown). The median of the horses age was 15 years (min. = 6y, max. = 29y). There were seven mares and six geldings. The sex, breed and age distribution of the horses were in line with the common group composition of private riding horses in Germany, which do not include stallions or breeding mares [23–25].

Wolves, other wildlife and birds (for more detail see below) were observed around and in the pastures. The pastures were within the habitat of a stable wolf pack KN (Knappenrode-Seenland pack), for which the alfa female was registered under the genotype code GW180f and the alfa male under the genotype code GW566m. The alfa pair reproduced annually with 5 pups in 2018/19, 10 pups in 2019/20, 8 pups in 2020/21, 7 pups in 2021/22, and 7 pups in 2022/23 [33]. Furthermore, we recorded badgers, raccoons, red foxes, raccoon dogs, beech martens, wild boar, hare, roe deer, and red deer, as well as birds such as cranes, snipes, and hooded crows. In addition, domestic cats, dogs, goats and the human management personnel were documented by the wildlife cameras [34] (Table 1).

**Table 1:** Data of total observation and comparative observation period. Group 1 data is shown with a green background, group 2 data with blue and the data recorded at the pathway with yellow. Total data counts are given in bold typing.

documentation type	numbers recorded			
	total observation	comparative observation period		
		January 01. 2022–23. Mach 2022		
	2015–2022	pasture 1	pasture 2	pathway
**total**	**213579**	**22434**	**7126**	**17077**
unknown releaser	202518	21463	5859	15965
releaser visible	11.061	971	1267	1112
day	3	114	148	23
dawn	162	3	0	39
night	1565	188	378	124
day or night	206.176	22.129	6.600	16.891
**wolf total**	**984**	**89**	**0**	**175**
1 wolf	581	75	0	86
2 wolves	61	7	0	11
3 wolves	38	0	0	12
4 wolves	8	0	0	7
5 wolves	17	0	0	0
6 wolves	6	0	0	0
7 wolves	2	0	0	0
wolf standing	117	3	0	1
wolf trotting	695	86	0	174
wolf running	3	0	0	0
wolf sniffing—sighting the pastures	25	0	0	0
wolf inside the pasture	9	0	0	0
wolf day	126	29	0	14
wolf night	759	57	0	122
wolf dawn	85	3	0	39
**horse total**	**2176**	**246**	**22**	**764**
horse grazing	2048	243	22	732
horse trotting	55	0	0	2
horse running	40	3	0	30
horse observing	1	0	0	0
horse at fence	24	0	0	0
**wildlife total**	**3151**	**633**	**1241**	**160**
badger	3	0	0	0
hare	1342	396	294	104
immature boar	1	0	0	0
mature boar	866	22	796	0
redfox	810	202	139	56
racoon dogs	11	3	2	0
roe deer	95	10	8	0
red deer	17	0	0	0
beech marten	1	0	0	0
racoons	3	0	2	0
**birds total**	**13**	**3**	**0**	**5**
crane	1	0	0	0
hooded crow	11	3	0	5
snipe	1	0	0	0
domestic goat	4	0	0	0
domestic dog	12	0	4	0
domestic cat	13	0	0	6
car	1	0	0	0
Person	4709	0	0	2

### Technical equipment

Six cameras (TN01, TN02, TN03, TN05, TN07 und TN09), four of the type Wildblick (TN01, TN02, TN03, TN05) and two of the type Secacam raptor (TN07 und TN09) were used to record horses and wildlife [34]. The cameras had highly sensitive movement sensors and infrared light and a PIR reach for 82ft/25m. The cameras PIR angle was set at 120°, the releasing time was set at 1 sec., and the LED flashes were turned off, because wolves may be sensitive to flashes and might avoid the camera after being flashed after their first visit at the location [35]. Camera TNO1 and TNO3 were placed at the pathway and recorded movements on the pathway between the pastures and at the fences of pasture 1 and 2. Camera TNO5, TNO7 and TNO9 observed movements at pasture 1 and camera TNO2 recorded occurrences at pasture 2 and the roadway.

### Experimental procedure

Wildlife cameras were fixed at the described positions of the pathway and the pastures in January 2015 (Fig 1). We recorded movements of animals, persons or the environment directly in the front of the 120° visual field of each particular camera day and night continuously [36]. The camera recordings were checked at four week intervals. Information about horses and animals and their movements at the two pastures and the pathway between the pastures (see for detail below) were noted on an excel sheet. Pictures which showed humans were registered on an excel sheet and immediately deleted.

Between 1 January 2022 and 23 March, the comparative observation period was conducted. In this time interval, the two horse groups at pasture 1 and 2 remained constant but differed in the distribution of the horses’ breeds, ages and sexes. Five ‘mixed-breed horses’ were assigned to pasture 1 and the group was comprised of one pony, two warmbloods and two draught horses. The median of the horses age was 8 years (min. = 6y, max. = 29y). There were four mares and one gelding (S1 Table).

Eight ‘heavy breed horses’ were assigned to pasture 2. They were comprised of three heavy warmbloods and four draught horses (for one the breed and age was unknown). The median of the horses age was 16 years (min. = 13y, max. = 22y). There were three mares and five geldings (S1 Table).

### Observation protocol

The management of the horses was provided with an observation protocol for recording cases of presumed or visible wolf–horse contact (S2 File). The person in charge of daily management of the horses was asked to record wolf presence, and behavioural and welfare reactions of the horses to wolf presence. In any such case the following should have been reported:

- the number and kind of domestic animals on the pasture,
- whether wolves attacked the horses,
- whether the horses fled from the pasture, and
- whether they damaged the fences.
- In addition, unusual behaviour in the horses such as
   I movements, trembling, sweating, and
   II aggressive behaviour towards dogs, as well as
- injuries in the horses.

### Data

Excel, version 2016, was used for the registration of the data. Between January 2015 and July 2022, 213579 camera recordings were checked. There were 5673 pictures taken during the day, 162 at dawn, 3 in the evening, 1565 during the night and for 206176 pictures, the day or nighttime was not recorded. In 202518 cases, in which the camera made a recording, just the usual natural surroundings of the camera were shown, without any trace of a person or animal who may have caused the release. In the remaining 11061 pictures, an animal or person were clearly visible and these were used for further analysis (termed ‘releaser visible’ in Tables 1 and S2).

For the comparative observation period (1 January 2022 to 23 March), as the horses at pasture1 and pasture 2 differed in breed, age and sex (S1 Table), we separated camera recordings for pasture 1, pasture 2 and the pathway between the pastures (Fig 1). At pasture 1, the cameras made 22434 recordings; for 21463 pictures the reason for the release was unknown while 971 pictures were useful for documenting animals on the pasture. The camera at pasture 2 made 7126 pictures, from which 5859 were without and 1267 with the releasing individual. From the pathway between the two pastures, we received 17077 recordings from which 15965 were without any sign of a releaser and 1112 showed the releaser. We used 971 pictures of pasture 1, 1267 pictures of pasture 2 and 1112 pictures of the pathway between the pastures for further analysis (Table 1).

### Camera protocol

We extracted the following information from the pictures, whenever the pictures were clear enough for drawing a conclusion (S1 File):

- location of the particular camera
- daytime of the camera recording
- kind of animal visible in the picture
- number of animals visible in the picture
- age status of the recorded animal (juvenile, subadult, adult)
- behaviour of the recorded animal

### Data analysis

The software R statistic (R Development Core Team, Boston MA, USA, 2021) with its package R commander was applied for data analysis, and Excel 2016 for visualizing the data (see complete raw data at S2 Table). The data were not normally distributed (Shapiro-wilks test: most p < 0.05). Binomial tests and Chi Square test were used for likelihood equations. The complete data of the comparative observation period were analysed with a Principal Component Analysis (PCA) for describing mutual and overlapping eigenvectors of the data. Because the PCA revealed a 99% proportion of variance in the first eigenvector of pasture 1, pasture 2 and pathway data, but a deviation of pasture 2 from pasture 1 and the pathway data in the second eigenvector, we continued to analyze such deeper layers of wildlife and wolf distributions on the pastures and the pathway by applying nested Generalized Linear Models. We applied the following formula: glm(formula = number_recordings ∼ location %in% recording_type, family = gaussian(identity), data = Dataset). The distribution of breeds, age and sex between the two horses groups was analyzed with the following formula glm(formula = group_num ∼ sex.num + breed_type_num + age, family = binomial(logit), data = Dataset). All factors under debate were fixed factors, set by the experiment. The significance level was set at 0.05 for all tests. All tests were two-tailed. Please see the complete statistical data at S3 File.

### Ethics statement

The regional animal welfare board of the county Bautzen confirmed that no animal welfare permission was needed. Because the study remained observational throughout, no pain, damage or suffering was caused to any animal throughout the observation period. According to the regulatory guidelines, the recordings of the wildlife cameras were documented on an excel sheet and directly deleted thereafter.

## Results

### Wildlife, wolf and horse documentation

Throughout the whole observation period (January 2015 to July 2022) no wolf attack on horses was recorded (Table 1). There was no case in which horses showed unusual behavior or body conditions from which a wolf attack could have been assumed. Therefore, the observation protocols for recording wolf attacks (S2 File) were not applied.

**Fig 2:**
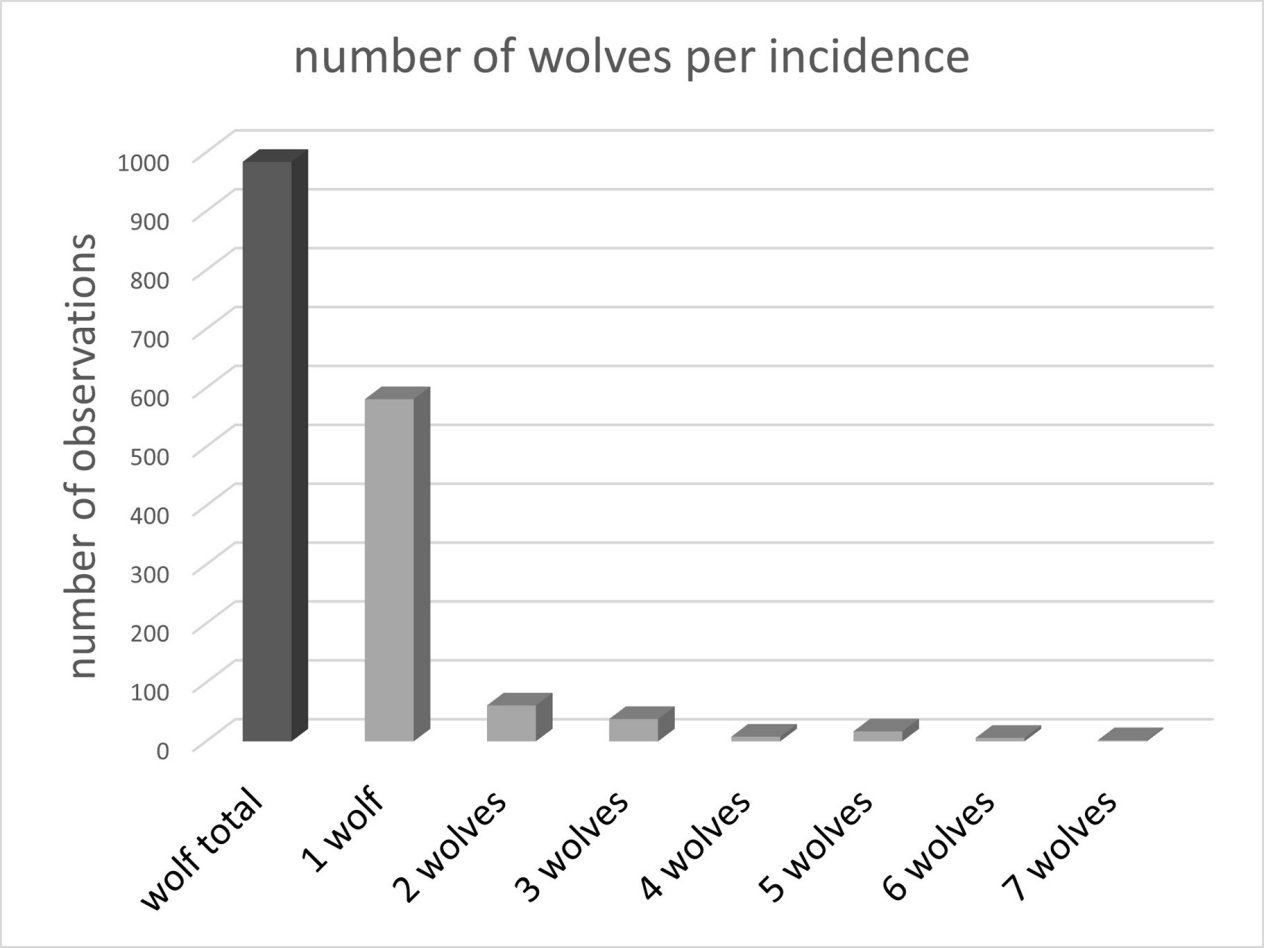
Wolves recorded between January 2015 and July 2022. The dark grey column shows the total number of wolves observed at the observation area. The light grey columns show the number of wolves that were observed together in any one incidence (Table 1).

Wolves were recorded in close proximity to or in the pastures in 984 pictures (Fig 2). Most pictures of wolves were conducted during the night (N = 759), only a few were taken at dawn (N = 85), some were made during the day (N = 126; Chi Square test: N = 970, df = 2, x² = 883.14, p < 0.001, Table 1).

**Fig 3:**
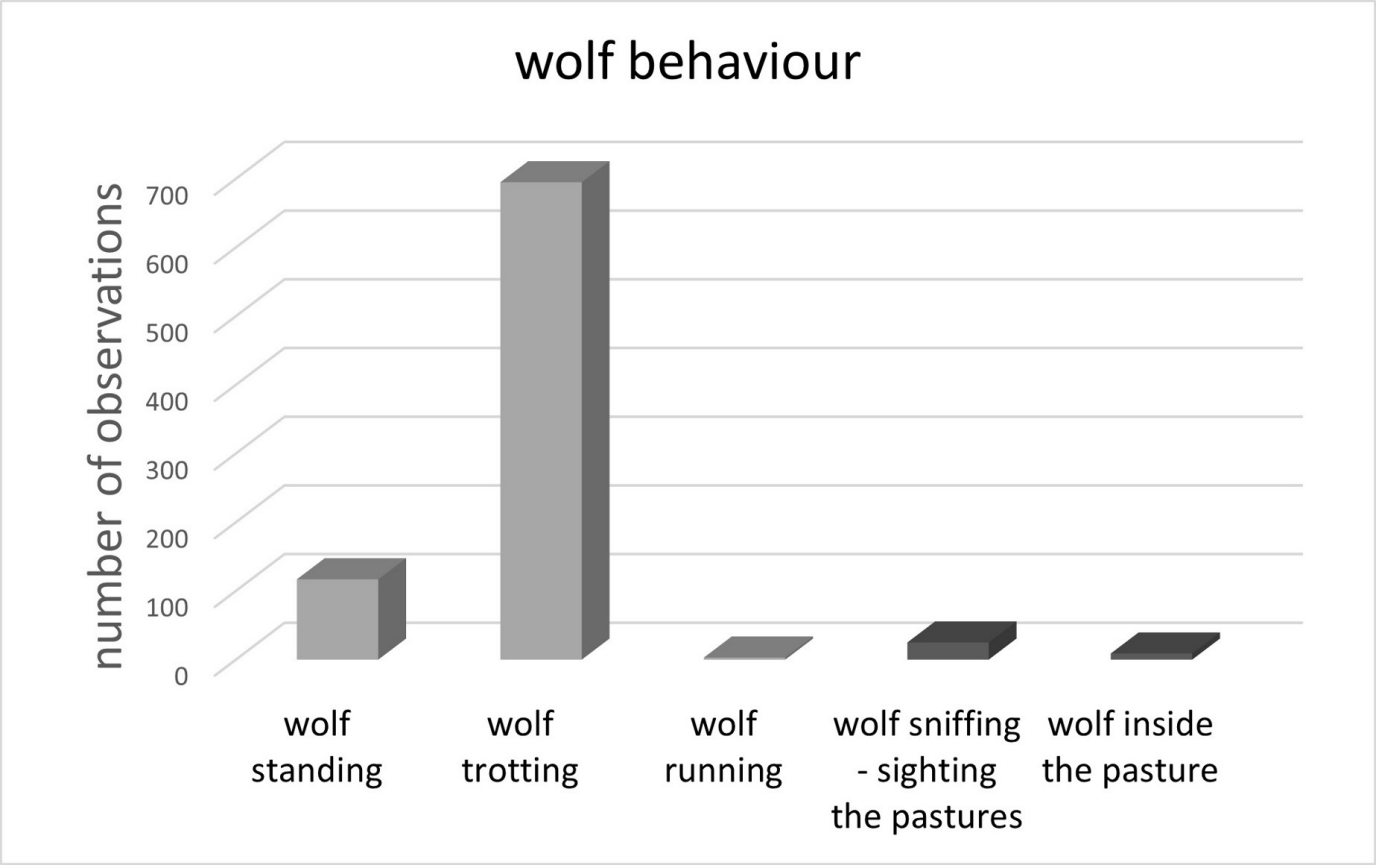
Wolf behaviour. The light grey columns visualize when wolves were recorded standing (N = 117), trotting (N = 695) and running (N = 3) along, inside and between horse pastures. The dark grey columns show when wolves were observing horse pastures (N = 25) and when wolves were recorded inside horse pastures (N = 9).

Most wolves were trotting along the fences (N = 695) some were standing (N = 117), some were sniffing towards the horses (N = 25) and a very few were either running outside the pasture (N = 3), or standing within the pasture (N = 9, Chi Square test: N = 849, df = 4, x^2^ = 2080.5, p < 0.001, Fig 3).

We also wrote down the number of wildlife documentations (N = 3151). The majority of wildlife consisted of hare (N = 1342), followed by wild boar (N = 867), red foxes (N = 810) and roe deer (N = 95, Chi Square test: N = 3114, df = 3, x^2^ = 1019.3, p < 0.001). In addition, we documented some red deer (N = 17) raccoon dogs (N = 11), hooded crows (N = 11), badgers (N = 3), raccoons (N = 3), a beech marten (N = 1), a crane (N = 1) and a snipe (N = 1). We also noted whether camera recordings showed domestic cats (N = 13), dogs (N = 12), goats (N = 4) and the human management personnel (N = 4709) (Table 1).

In addition, we extracted 2176 cases in which horse behaviour was reported (Tables 1 and S2). In the majority of cases (N = 2048) horses were grazing, in N = 24 cases horses stood at the fence, in one case (N = 1) a horse appeared to observe a situation outside the fence, N = 55 pictures showed horses which were moving and at N = 40 pictures we found horses were running (Chi Square test: N = 2168, df = 4, x^2^ = 7571.2, p < 0.001). For the cases in which horses were running, we confirmed whether wolf presence was recorded on the particular day (Table 2). The 40 pictures of running horses were taken on six days. On three of the days no wolf was reported, on two days one wolf was recorded in 3 pictures each, and on another day one wolf was recorded in 5 pictures.

**Table 2:** Observations on days when horses were recorded running. Wolf data are presented with a grey and wildlife data with a brown background.

running horse—date	wolfs recorded	wildlife recorded
30.04.2021	3 x 1 adult wolf	8 hare, 8 red foxes, 5 roe deer, 5 wild boars, 1 crow, 2 racoon dogs, 2 cats
26.10.2021	0	2 hare, 2 red fox
04.01.2022	3 x 1 adult wolf	0
10.02.2022	0	1 racoon dog, 1 red fox
11.02.2022	5 x 1 adult wolf	0
02.06.2022	0	0

### Wolf and wildlife presence at the pastures of horse groups 1 and 2

The group of horses in pasture 1 was comprised of young horses (median age = 8 years) of mixed breed, with one male and four mares, whereas the horses of group 2 were older (median age = 15.5 years), of heavy horse breeds, with five males and three mares. While the two groups did not significantly differ in their age and breed (GLM: p > 0.1), group 1 tended to have more mares but fewer males than group 2 (GLM: N = 13, z = 1.649, p = 0.099, S1 Table).

Generally, the data patterns at the pasture of the two horse groups and the pathway matched each other between 1 January 2022 and 23 March 2022. The PCA analysis proved a 99% overlap in the variance of the first component / eigenvector when comparing between the total data of pasture 1, pasture 2 and the pathway (PCA Comp1, comp loadings: pasture 1 = 0.578, pasture 2 = 0.575, pathway = 0.578, Comp1 variance = 2.981, SD = 1.7, proportion of variance = 0.994, S3 File).

Similarly, for the total data of pasture 1, pasture 2 and the pathway, the majority of recordings did not show the identity of the releaser (GLM: N = 46637, group1: t = 6.453, p < 0.001, pathway: t = 4.911, p < 0.001, group2: t = 2.045, p = 0.044). However, similar numbers of recordings clearly visualized the releaser for pasture 1 (N = 971), for pasture 2 (N = 1267) and at the pathway (N = 1112) (GLM: N = 46637, all p > 0.05, S3 File).

Furthermore, the PCA analysis revealed that a small proportion of the data of pasture 2 significantly deviated from the data of pasture 1 and the pathway (PCA Comp2, comp loadings: pasture 1 = 0.391, pasture 2 = -0.817, pathway = 0.423, Comp2 variance = 0.0017, SD = 0.13, proportion of variance = 0.006; S3 File).

**Fig 4:**
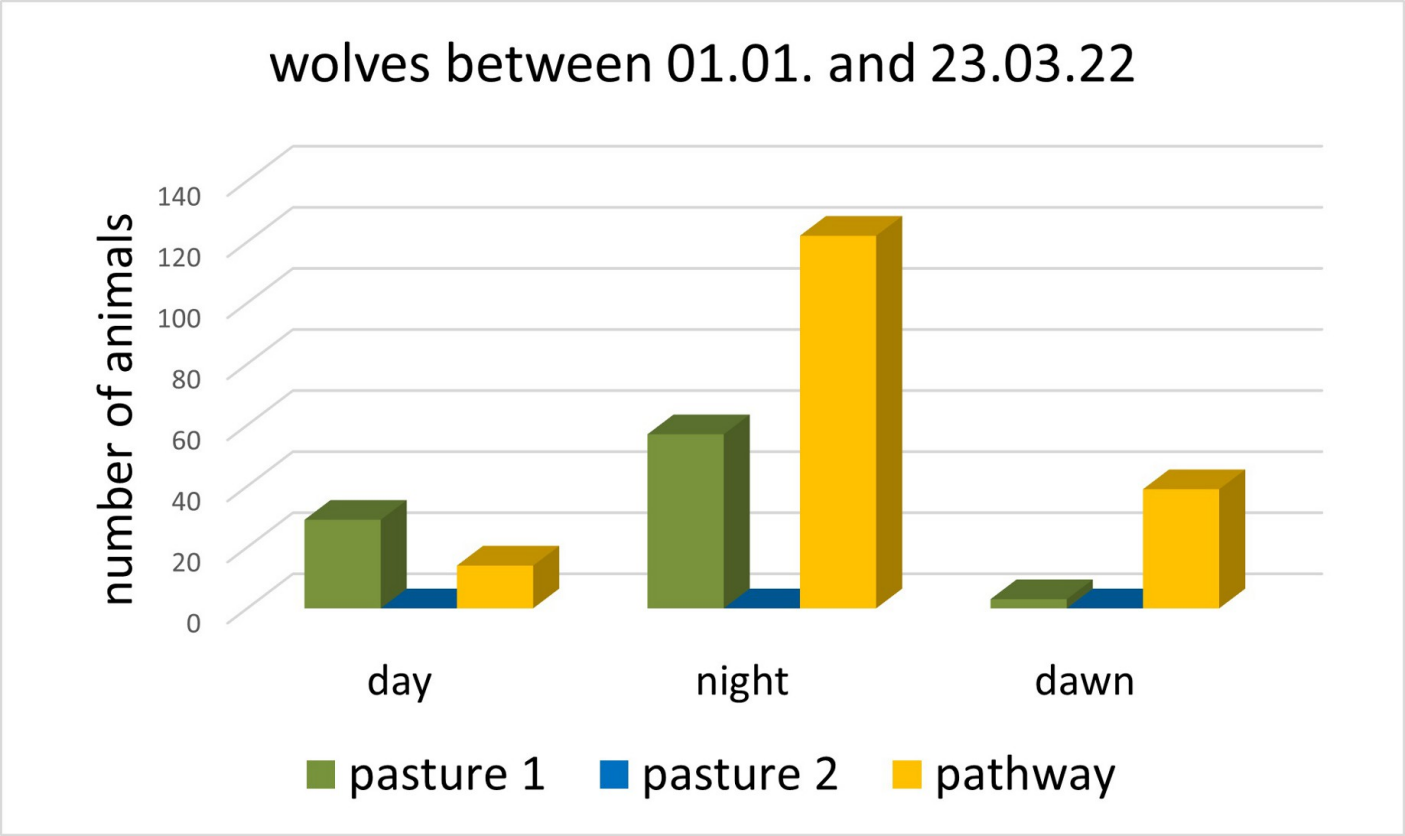
Wolves recorded close to or at pasture 1, pasture 2 and the pathway at the comparative observation. No wolf was recorded at pasture 2 between the 1^st^ January and the 23^rd^ March 2022, neither during the day, the night, or the dawn (Table 1).

Firstly, most pictures of wolves were taken in the pathway (N = 175, GLM: N = 264, t = 4.045, p < 0.001) and pasture 1 (N = 89, GLM: N = 264, t = 1.849, p = 0.07), whereas there were no recordings of wolves taken in pasture 2 (N = 0). The movement speeds and numbers of wolves did not differ significantly between those recorded in the pathway and pasture 1 (GLM: N = 264, all p > 0.05, Table 1 and Fig 4).

Secondly, the pictures were mostly taken at night in the pathway (GLM: N = 46637, t = 2.023, p = 0.046) and for pasture 1 (GLM: N = 46637, t = 2.659, p = 0.009), whereas there was no significant difference between the numbers of recordings made at particular daytimes for pasture 2 (GLM: N = 46637, t = 0.839, p = 0.4, Table 1 and Fig 4).

**Fig 5:**
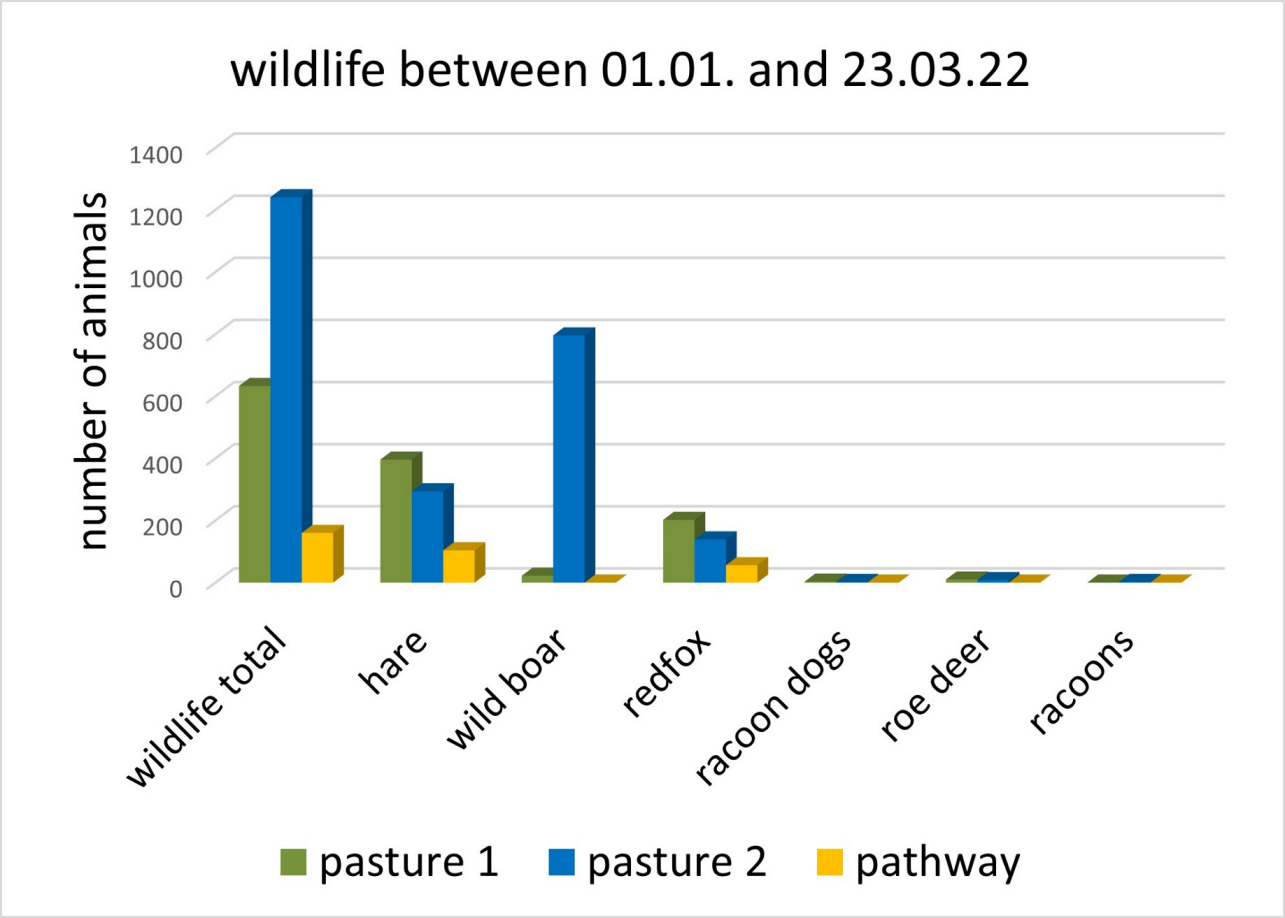
Wildlife recorded at particular pastures and the pathway during the comparative observation. Columns of dark color show the total number of wildlife, columns of light color the specific wildlife species recorded. While wildlife on pasture 1 was mostly hare (63%), wildlife on pasture 2 showed mostly wild boar (64%) (Table 1, Chi square test: both p < 0.001).

Thirdly, most wildlife was recorded at pasture 2 (N = 1241, GLM: N = 2034, t = 7.722, p < 0.001). Furthermore, wildlife was more frequent at pasture 1 (N = 633, GLM: N = 2034, t = 3.889, p < 0.001) than on the pathway (N = 160). Wildlife documentation at pasture 1 (N = 633) was comprised mostly of hare (N = 396, 63%), followed by red foxes (N = 202, 32%), wild boar (N = 22, 3.5%), roe deer (N = 10, 1.6%) and raccoon dogs (N = 3, 0.5%) (Chi square test: N = 633, x^2^ = 932.66, df = 4, p < 0.001, Table 1 and Fig 5). At pasture 2, wildlife (N = 1241) was comprised mostly of wild boar (N = 796, 64%), followed by hare (N = 294, 24%), red fox (N = 139, 11%), roe deer (N = 8, 0.6%), raccoons (N = 2 (0.2%), and raccoon dogs (N = 2, 0.2%) (Chi square test: N = 1241, x^2^ = 2334.1, df = 5, p < 0.001, Table 1 and Fig 5).

## Discussion

Wolves were frequently documented close to horse pastures in the present long-term field study, which was conducted between January 2015 and July 2022. Sometimes wolves were recorded within the pastures. Throughout the whole observation period, we recorded no wolf attack on horses, while this has frequently been reported from wolves in the East and the South of Europe [6–11]. Furthermore, the horses did not display any signs of reduced welfare or panic [2, 3, 27, 29] in the presence of wolves. The recorded running of horses at six days may have been caused by various disturbances. Wolves were recorded only on three of the six days, further reasons for the horses’ restlessness may have been wildlife presence, insect loads or sounds produced by the human environment [28].

The observations of the present study may support a previous assumption, that Central European horses may not have lost their abilities to deal with wolves that do not attack, even though they did not experience predator contact in the past centuries [27–30]. It confirms observations in natural settings, in which a coexistence of equids and non-hunting predators was frequently observed [37]. However, the present observational study can,not conclude on the horses’ emotional stage and welfare at wolf contact. Future approaches need to combine wildlife documentations with additional parameters for measuring horse emotionality and welfare [38–43].

During the comparative observation period, the two pastures were grazed by stable horse groups. While wolves were recorded for pasture 1 with one gelding and four mares of, insignificantly, younger age and various breeds, no wolf was recorded for pasture 2, with five geldings and two mares of, insignificantly, older age and heavy horse breeds. The wolves may have avoided the group with five geldings, as geldings may protect their group members against predators and other dangers as reported for stallions [20, 21]. However, future studies need to focus on comparing sex with breed, age and group size effects on horse anti-predator behaviour.

Breeds may differ in their anti-predator behaviour [3, 13], as shown for Arabian and Konik horses [29, 30]. Arabian horses were reported to respond strongest to Arabian leopard growl (*Pantera pardus nimr*) by gathering in one line and approaching the growl playback. Konik horses responded stronger to wolf howl (*Canis lupus*) by reducing individual distances in their groups and retreating from the location of the wolf howl [30]. However, it remains to be seen whether Thoroughbred horses are just more careful and sensitive in danger recognition [29, 30] and whether they would actually defend themselves from predators’ attacks. In the present study heavy horse breeds were not confronted with wolves, but also warmblood type horses were not attacked and we did not record any anti predatory response in horses.

Also, the age of horses may be decisive. In northern Spain, Portugal and Italy foals were preferentially attacked by predators right after birth and around the age of 1 when their dams were busy with new offspring [8, 9, 13]. Foals were not part of the present study.

Furthermore, allowing horses to form stable groups which include breeding animals such as mares with offspring and stallions may be very important [23–25]. When mares protected their foals, they were said to take the foals in their middle and approach the wolves [8]. Wolves usually retreated. They remained waiting, close to the horses, and started an attack only when offspring separated from the group. Generally, wolves avoided mature horses and they fled when humans intervened [8, 9, 13]. Only in rare cases of severe wolf pack attacks do the horses flee, galloping in a straight line [8].

Finally, some horses form stable social relationships, so called social bonds, with other group members [20, 44]. Social relationships are of high value in horses, as they help to mutually protect offspring [45] and because horses learn from one another [46, 47]. Horses even protect their social relationships by intervening in friendly [48] and aggressive interaction of group members [49] and by showing conflict resolution behaviour [50]. However, about 15% of mature horses and 90% of the maturing offspring appear to be only loosely bonded and change between the subgroups of their herds [21, 22]. Mares which changed groups were more likely to lose foals [13], whereas, offspring of bonded mares [45] and of medium sized, stable harem groups, numbering up to about 10 animals, were reported to have better survival options [13].

Wolves may avoid larger horse group [13]. Therefore, the best conditions for predator protection might be given in medium sized, stable horse groups, which include breeding animals such as breeding mares and stallions, and in which members form stable social relationships [3, 13].

Furthermore, the wolves may have avoided the risk of hunting horses and may have preyed on easily accessible prey [11], as plenty of wildlife was recorded at the pastures. In addition, they may have avoided hunting wild boar at pasture 2, but may have hunted hare and roe deer at the pasture 1, which are huntable with less danger and are, therefore, preferred prey for wolves [11, 18, 19]. Both the hunting activities of wolves and the documentation of preferred prey for wolves may have resulted in more camera recordings taken at night at pasture 1 and the pathway, than at pasture 2.

Even though it appears to be difficult to record animal welfare parameters during long-term, field studies [38] and when horses are in contact with wolves [28], future studies should aim to include animal welfare measures that allow for evaluating the horses’ emotionality. Enhanced methods and technical equipment for precise behaviour recordings may offer confirmation on whether Central European geldings respond differently to wolves than stallions and breeding mares or whether the horses’ breed, age and experience is more decisive [3, 13, 29, 30]. Enhanced techniques may also provide fewer camera recordings with unrecognizable releasers, which may potentially have resulted in underrepresented recordings of animals at high movement speed at the present study.

Finally, it needs to be documented, that even though wolf attacks on horses have been reported and discussed in Germany since 2016, only a few horse owners changed their horse management strategies or established predator protection measures [3]. It should be carefully monitored whether wolves in Germany start specializing on hunting horses, as reported from other countries [7, 9, 12, 13]. For preventing wolf predation on horses, it may be advisable to install wolf deterrent fences [32]. Furthermore, foaling mares, foals and small ponies should be protected by human presence, for example, by returning them to pastures, equipped with wolf deterrent fences, close to human housing for the night [13, 28, 31]. Finally, horse owners may consider training guard dogs, which were frequently reported to be very helpful in predator protection for many species, also horses [28, 51].

## Conclusion

This is the first long-term, field study which documents wolves close to and at horse pastures in Central Europe. Wolves did not attack the mature horses and the horses did not respond to the presence of wolves with signs of reduced welfare or panic.

## Supporting information

S1 Table Data of the horses at the comparative observation period. (green = group 1, blue = group 2).
(PDF)

S2 Table Raw data of horse, wolf and wildlife observations. List of complete camera recordings made between January 2015 and July 2022.
(XLSX)

S1 File Wildlife camera recordings showing wolves close to the horses’ pastures. (PDF)

S2 File Observation protocol for horse management. (PDF)

S3 File Statistical data. Complete models and test results of statistical analyses.
(PDF)
