# Supplementary material for: Wolf contact in horses at permanent pasture in Germany
Source: PLoS One. 2023 Aug 10;18(8):e0289767. doi: 10.1371/journal.pone.0289767 (PMC10414631; doi:10.1371/journal.pone.0289767)
Supplement: S1 Table — (green = group 1, blue = group 2). (PDF) [file pone.0289767.s001.pdf]

S1 Table. Data of the horses at the comparative observation period.

(green = group 1, blue = group 2).

horse name	group	sex	breed	age
Cordi	group1	f	warmblood	29
Montana	group1	f	pony	8
Joe	group1	m	draught horse	4
Milly	group1	f	warmblood	4
Donna	group1	f	draught horse	22
Cuno	group2	m	heavy warmblood	17
Melchor	group2	m	heavy warmblood	17
Elton	group2	m	draught horse	15
Erbse	group2	f	draught horse	15
Poly	group2	f	heavy warmblood	16
Dann	group2	m	NA	NA
Jack	group2	m	draught horse	13
