# Supplementary figures and images for: Wolf contact in horses at permanent pasture in Germany
Source: PLoS One. 2023 Aug 10;18(8):e0289767. doi: 10.1371/journal.pone.0289767 (PMC10414631; doi:10.1371/journal.pone.0289767)

**S1 File. Wildlife camera recordings showing wolves close to the horses' pastures**

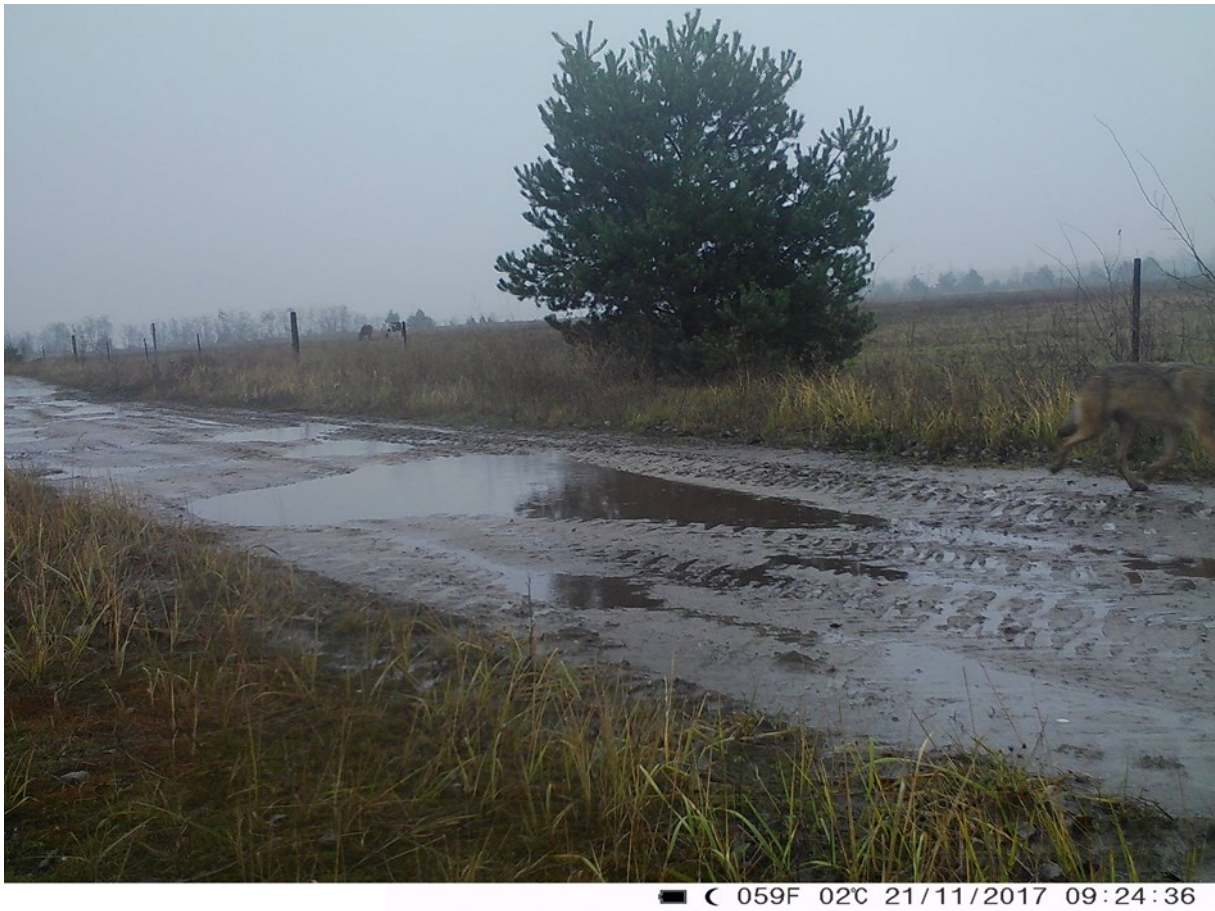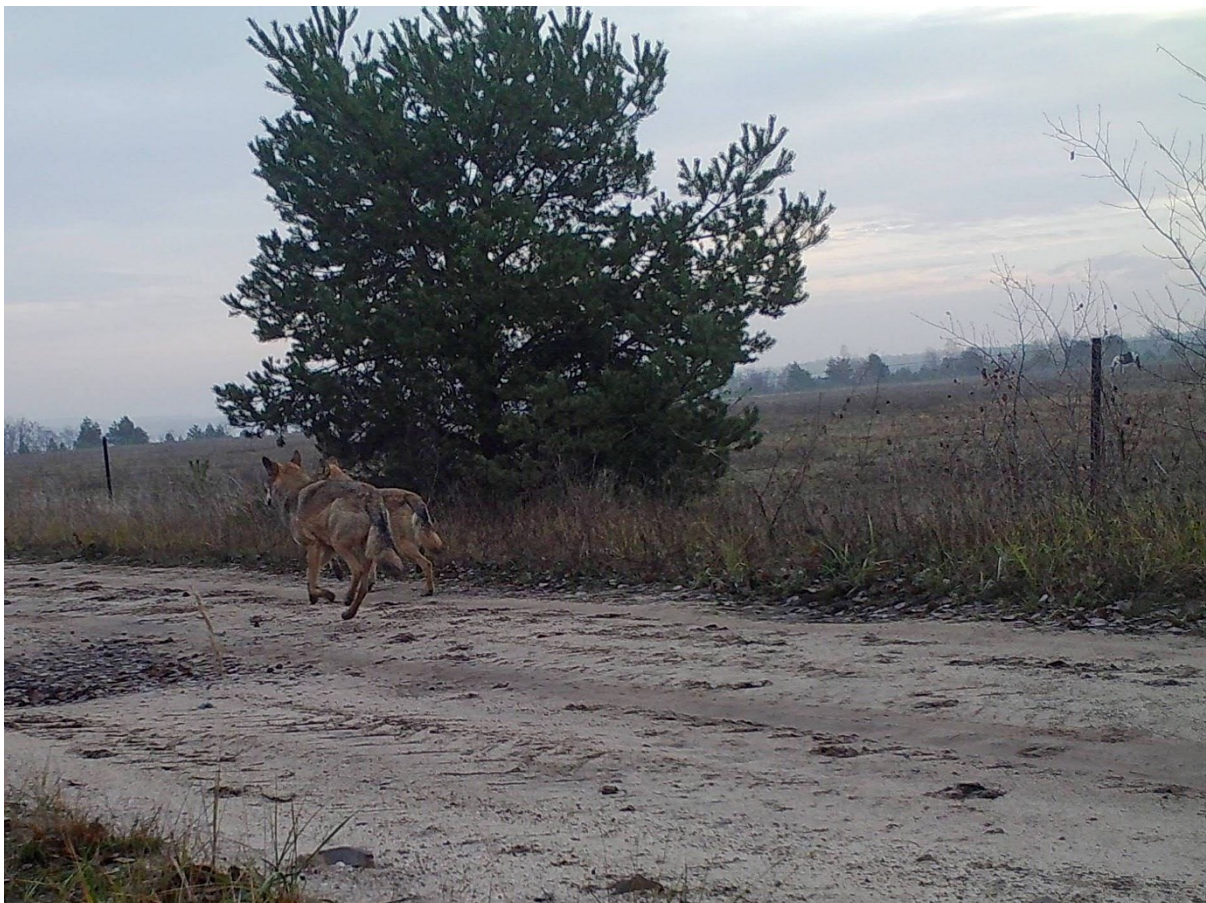

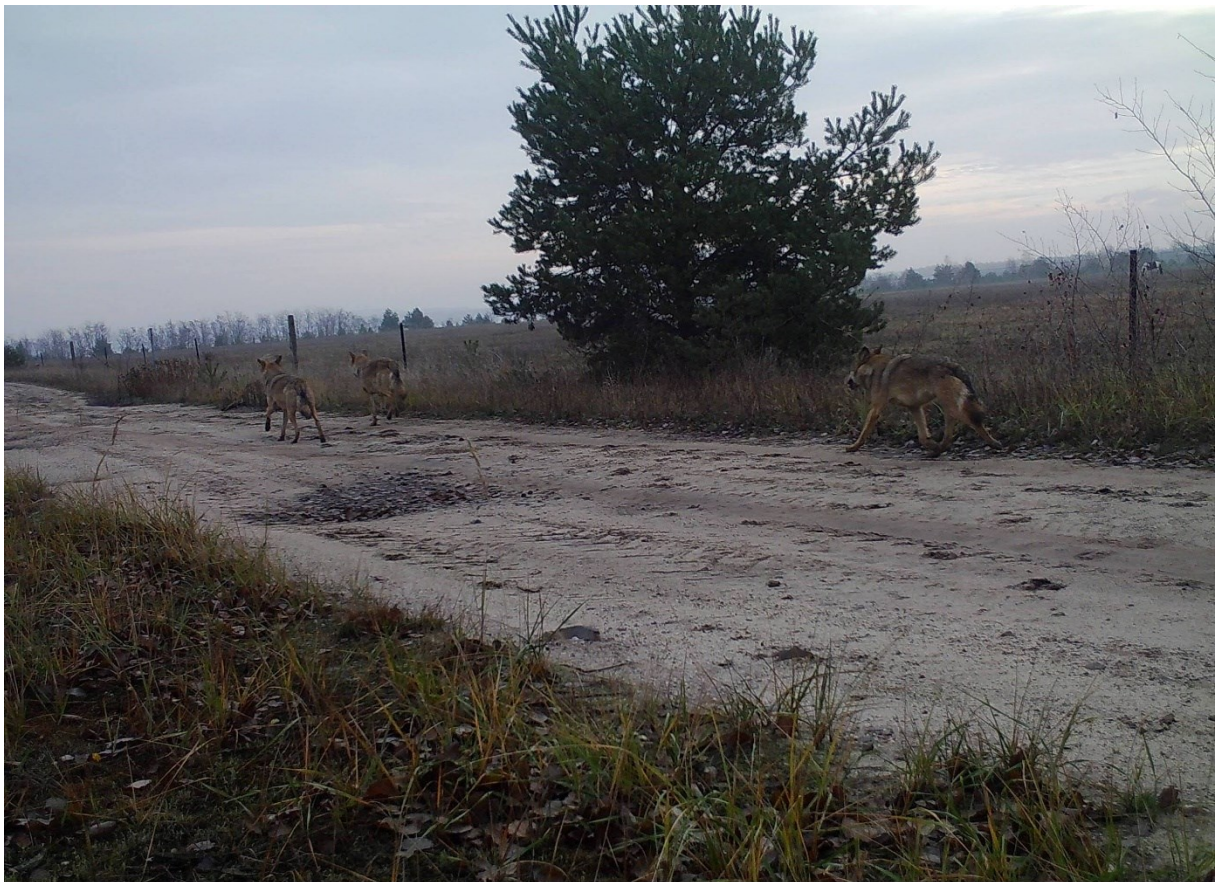

● 056F 01°C 24/11/2018 08:30:22

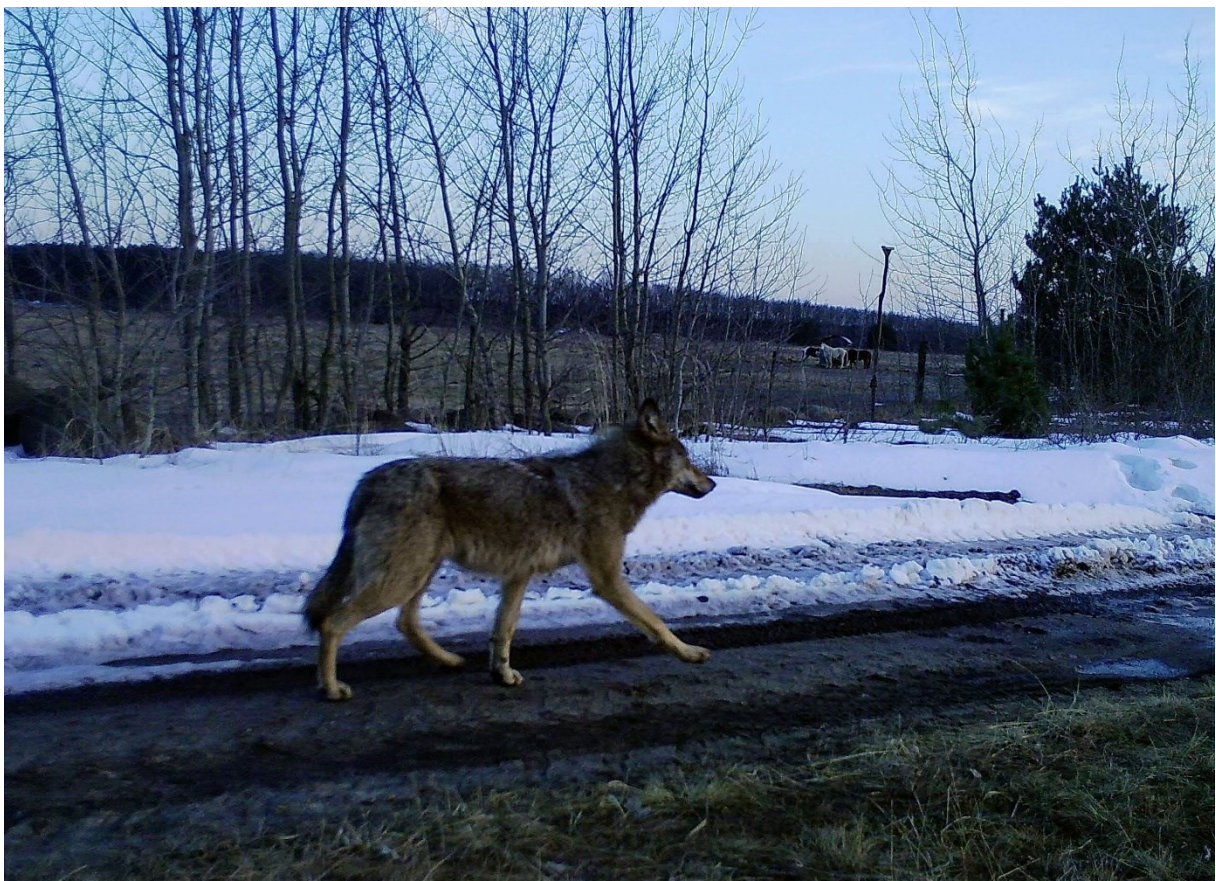

☾ 05C Mi 21.03.2018 17:58:52

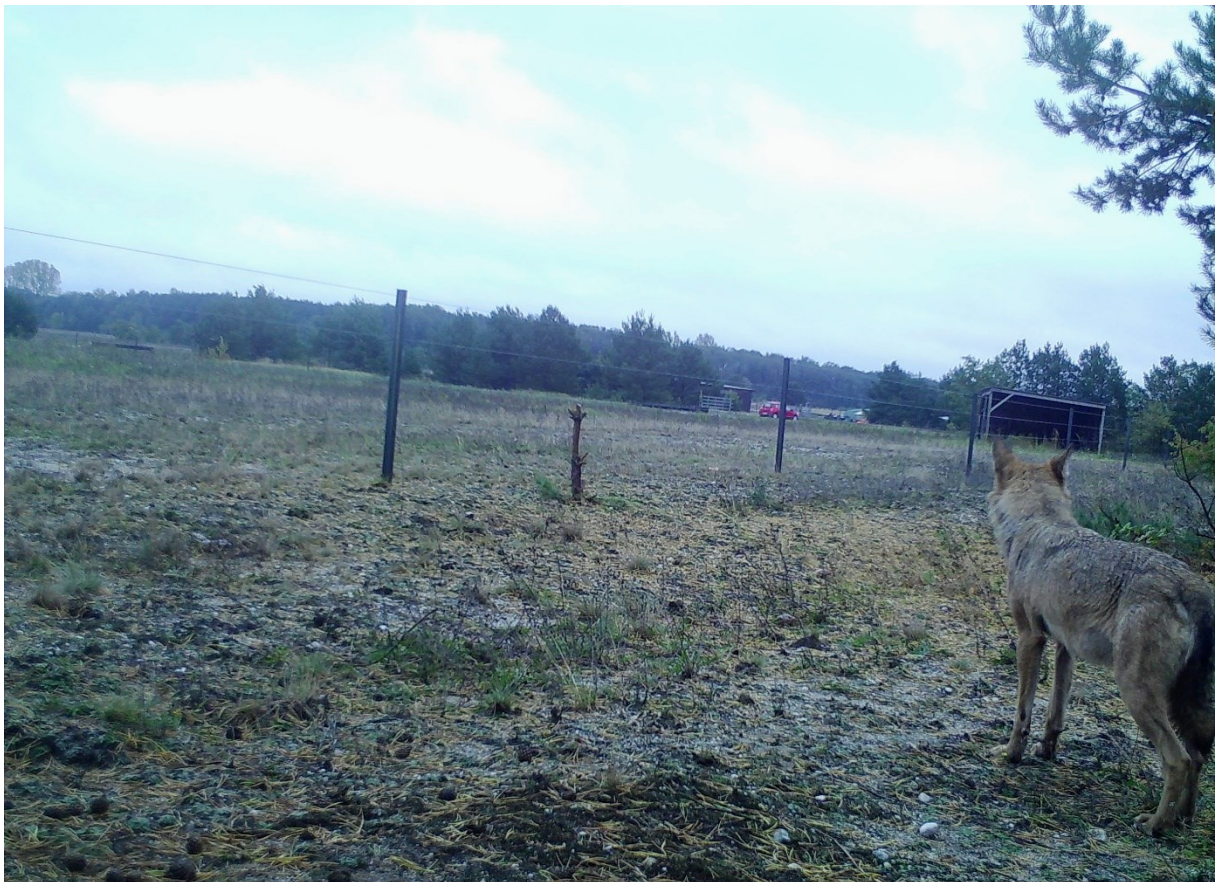

● 074F 11°C 08/10/2017 07:47:15

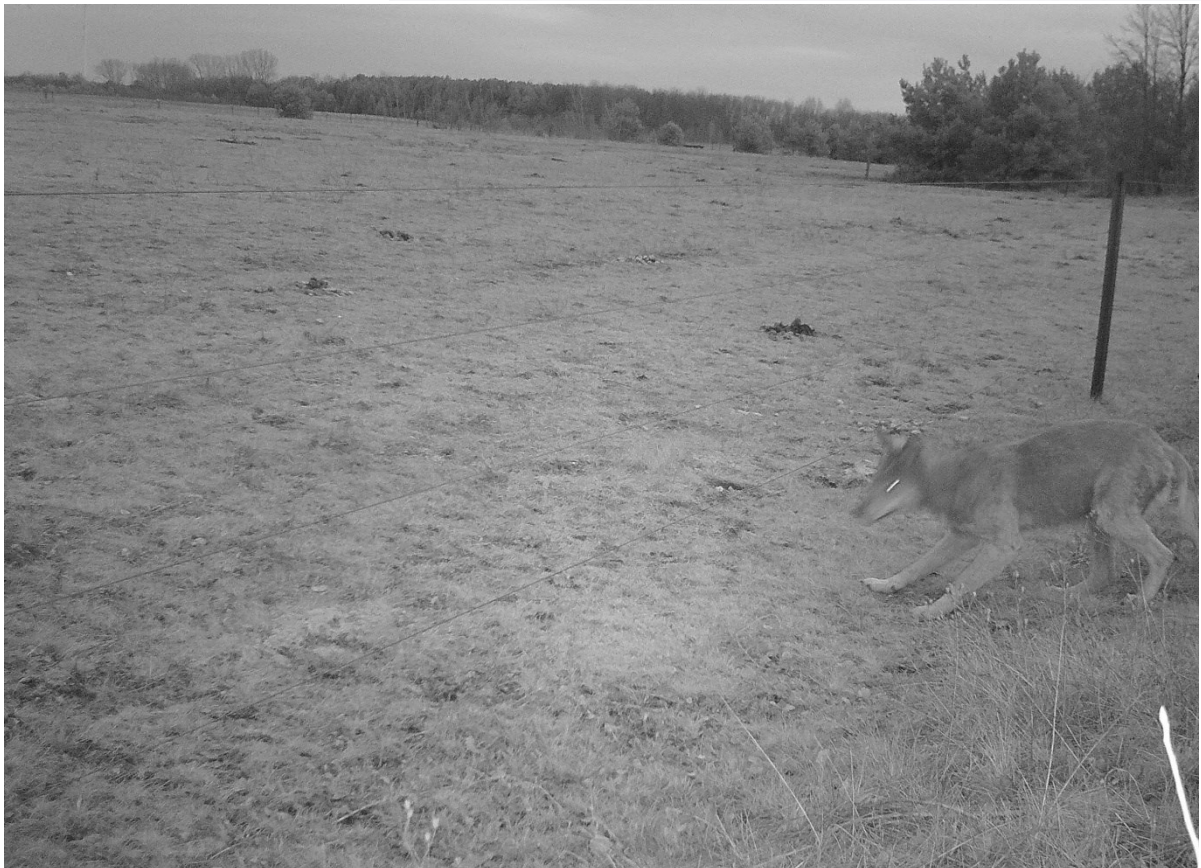

► 16C So 11.03.2018 18:17:51

Supplement: S1 File — (PDF) [file pone.0289767.s003.pdf]
