# Supplementary material for: Wolf contact in horses at permanent pasture in Germany
Source: PLoS One. 2023 Aug 10;18(8):e0289767. doi: 10.1371/journal.pone.0289767 (PMC10414631; doi:10.1371/journal.pone.0289767)
Supplement: S2 File — (PDF) [file pone.0289767.s004.pdf]

"VFWPW" Dokumentation Pferdeverhalten

Bearbeiter:innen

Datum:

Untersuchungsgebiet: Terra Nova / Räber / Rodewald

Uhrzeit: bis

Beschreibung (was ist vorgefallen?):

Herdenbildung? etablierte Gruppe?

Weitere Weidetiere anderer Arten auf gleicher Weide/in der Nähe? Schaf, Ziege, Esel, Rind(Mutterkühe)

Wie haben Pferde auf Wolfskontakt reagiert?

Gab es Auffälligkeiten, welche?

Indikatoren für Verhalten(szustand) der Pferde: Ohrstellung,

Lautäußerungen,

Bewegungsverhalten, Schwitzen, Zittern?

Ausgebrochen:

Zäune :intakt? defect/zertört?

Tiere verletzt:

Schweißgebadet:

Aggressiv/besonders aufmerksam gegen Hunde:

Nachweis Wolfspräsenz: ☐ **Sichtungen** ☐ **Heulen** ☐**Trittsiegel** ☐ **Kamerabilder**

Andere Hinweise:

Wann war die letzte Tierkontrolle?
