# Supplementary material for: Wolf contact in horses at permanent pasture in Germany
Source: PLoS One. 2023 Aug 10;18(8):e0289767. doi: 10.1371/journal.pone.0289767 (PMC10414631; doi:10.1371/journal.pone.0289767)
Supplement: S3 File — Complete models and test results of statistical analyses. (PDF) [file pone.0289767.s005.pdf]

S3 File. Statistical Data

Principal Component Analysis (PCA)

Wolf contact in horses at permanent pasture in Germany

```
Rcmdr+    PCA <- princomp(~pasture1+pasture2+pathway, cor=TRUE, data=Dataset)
Rcmdr+    cat("\nComponent loadings:\n")
Rcmdr+    print(unclass(loadings(.PC)))
Rcmdr+    cat("\nComponent variances:\n")
Rcmdr+    print(.PC$sd^2)
Rcmdr+    cat("\n")
Rcmdr+    print(summary(.PC))

Component loadings:
            Comp.1     Comp.2      Comp.3
pasture1 0.5782279  0.3911357  0.71600654
pasture2 0.5757171 -0.8174422 -0.01838649
pathway  0.5781023  0.4228488 -0.69785139

Component variances:
      Comp.1       Comp.2       Comp.3
2.9819075241 0.0174284852 0.0006639907

Importance of components:
                          Comp.1      Comp.2       Comp.3
Standard deviation     1.7268201 0.132016988 0.0257680171
Proportion of Variance 0.9939692 0.005809495 0.0002213302
Cumulative Proportion  0.9939692 0.999778670 1.0000000000
```

Generalized linear Models (nested, fixed factors)

Total Data

```
Call:
glm(formula = number_recordings ~ location %in% recording_type, 
    family = gaussian(identity), data = Dataset)

Deviance Residuals: 
     Min        1Q    Median        3Q       Max  
-13985.0     -41.3      -1.7       0.0   16520.5  

Coefficients: (1 not defined because of singularities)
                                                Estimate Std. Error t value      Pr(>|t|)    
(Intercept)                                      16.5714  1267.9637   0.013      0.98960    
locationgroup 2:recording_typebird              -16.5714  3586.3428  -0.005       0.99632    
locationgroup1:recording_typebird               -13.5714  3586.3428  -0.004       0.99699    
locationpathway:recording_typebird              -11.5714  3586.3428  -0.003       0.99743    
locationgroup 2:recording_typebird_species      -16.5714  2314.9743  -0.007       0.99430    
locationgroup1:recording_typebird_species       -15.5714  2314.9743  -0.007       0.99465    
locationpathway:recording_typebird_species      -14.9048  2314.9743  -0.006       0.99488    
locationgroup 2:recording_typecat               -16.5714  3586.3428  -0.005       0.99632    
locationgroup1:recording_typecat                -16.5714  3586.3428  -0.005       0.99632    
locationpathway:recording_typecat               -10.5714  3586.3428  -0.003       0.99765    
locationgroup 2:recording_typedaytime          1764.9286  2102.6798   0.839       0.40335    
locationgroup1:recording_typedaytime           5591.9286  2102.6798   2.659       0.00917 ** 
locationpathway:recording_typedaytime          4252.6786  2102.6798   2.023       0.04591 *  
locationgroup 2:recording_typedog               -12.5714  3586.3428  -0.004       0.99721    
locationgroup1:recording_typedog                -16.5714  3586.3428  -0.005       0.99632    
locationpathway:recording_typedog               -16.5714  3586.3428  -0.005       0.99632    
locationgroup 2:recording_typegeneral          4734.0952  2314.9743   2.045       0.04359 *  
locationgroup1:recording_typegeneral          14939.4286  2314.9743   6.453 0.00000000445 ***
locationpathway:recording_typegeneral         11368.0952  2314.9743   4.911 0.00000372017 ***
locationgroup 2:recording_typehorse               5.4286  3586.3428   0.002       0.99880    
locationgroup1:recording_typehorse              229.4286  3586.3428   0.064       0.94912    
locationpathway:recording_typehorse             747.4286  3586.3428   0.208       0.83535    
locationgroup 2:recording_typehorse movement    -12.1714  1964.3208  -0.006       0.99507    
locationgroup1:recording_typehorse movement      32.6286  1964.3208   0.017       0.98678    
locationpathway:recording_typehorse movement    136.2286  1964.3208   0.069       0.94485    
locationgroup 2:recording_typeperson            -16.5714  3586.3428  -0.005       0.99632    
```

locationgroup1:recording_typeperson	-16.5714	3586.3428	-0.005	0.99632
locationpathway:recording_typeperson	-14.5714	3586.3428	-0.004	0.99677
locationgroup 2:recording_typewildlife	1224.4286	3586.3428	0.341	0.73354
locationgroup1:recording_typewildlife	616.4286	3586.3428	0.172	0.86389
locationpathway:recording_typewildlife	143.4286	3586.3428	0.040	0.96818
locationgroup 2:recording_typewildlife_species	107.5286	1653.2223	0.065	0.94828
locationgroup1:recording_typewildlife_species	46.7286	1653.2223	0.028	0.97751
locationpathway:recording_typewildlife_species	-0.5714	1653.2223	0.000	0.99972
locationgroup 2:recording_typewolf	-16.5714	3586.3428	-0.005	0.99632
locationgroup1:recording_typewolf	72.4286	3586.3428	0.020	0.98393
locationpathway:recording_typewolf	158.4286	3586.3428	0.044	0.96486
locationgroup 2:recording_typewolf daytime	-16.5714	2314.9743	-0.007	0.99430
locationgroup1:recording_typewolf daytime	13.0952	2314.9743	0.006	0.99550
locationpathway:recording_typewolf daytime	41.7619	2314.9743	0.018	0.98564
locationgroup 2:recording_typewolf movement	-16.5714	1964.3208	-0.008	0.99329
locationgroup1:recording_typewolf movement	1.2286	1964.3208	0.001	0.99950
locationpathway:recording_typewolf movement	18.4286	1964.3208	0.009	0.99253
locationgroup 2:recording_typewolf_number	-16.5714	1793.1714	-0.009	0.99265
locationgroup1:recording_typewolf_number	-4.8571	1793.1714	-0.003	0.99784
locationpathway:recording_typewolf_number	NA	NA	NA	NA

Data Wolf

```
Call:
glm(formula = recordings_wolf ~ location_wolf %in% wolf_recording_classes, family = gaussian(identity), data = Dataset
)

Deviance Residuals:
    Min       1Q   Median       3Q      Max
-44.333  -16.571   -0.333    0.000  139.000

Coefficients: (1 not defined because of singularities)
                                                        Estimate Std. Error t value Pr(>|t|)
(Intercept)                                               16.571     13.848   1.197 0.239258
location_wolfgroup 2:wolf_recording_classeswolf          -16.571     39.168  -0.423 0.674750
location_wolfgroup1:wolf_recording_classeswolf            72.429     39.168   1.849 0.072657 .
location_wolfpathway:wolf_recording_classeswolf          158.429     39.168   4.045 0.000264 ***
location_wolfgroup 2:wolf_recording_classeswolf daytime  -16.571     25.283  -0.655 0.516351
loaction_wolfgroup1:wolf_recording_classeswolf daytime    13.095     25.283   0.518 0.607662
location_wolfpathway:wolf_recording_classeswolf daytime   41.762     25.283   1.652 0.107276
location_wolfgroup 2:wolf_recording_classeswolf movement -16.571     21.453  -0.772 0.444893
location_wolfgroup1:wolf_recording_classeswolf movement    1.229     21.453   0.057 0.954649
location_wolfpathway:wolf_recording_classeswolf movement  18.429     21.453   0.859 0.396018
location_wolfgroup 2:wolf_recording_classeswolf_number   -16.571     19.584  -0.846 0.403047
location_wolfgroup1:wolf_recording_classeswolf_number     -4.857     19.584  -0.248 0.805532
loaction_wolfpathway:wolf_recording_classeswolf_number        NA         NA      NA       NA
```

Data Wildlife

```
glm(formula = recordings_wildlife ~ location_wildlife %in% wildlife_recording_classes, family = gaussian(identity), da
ta = Dataset)

Deviance Residuals:
   Min      1Q  Median      3Q     Max
-124.1   -63.3   -16.0     0.0   671.9

Coefficients: (1 not defined because of singularities)
                                                                    Estimate Std. Error t value     Pr(>|t|)
(Intercept)                                                            16.00      47.83   0.335      0.74011
location_wildlifegroup 2:wildlife_recording_classesbird               -16.00     158.64  -0.101      0.92027
location_wildlifegroup1:wildlife_recording_classesbird                -13.00     158.64  -0.082      0.93518
location_wildlifepathway:wildlife_recording_classesbird               -11.00     158.64  -0.069      0.94514
location_wildlifegroup 2:wildlife_recording_classesbird_species       -16.00      99.57  -0.161      0.87332
location_wildlifegroup1:wildlife_recording_classesbird_species        -15.00      99.57  -0.151      0.88117
location_wildlifepathway:wildlife_recording_classesbird_species       -14.33      99.57  -0.144      0.88641
location_wildlifegroup 2:wildlife_recording_classeswildlife          1225.00     158.64   7.722 0.0000000068 ***
location_wildlifegroup1:wildlife_recording_classeswildlife            617.00     158.64   3.889      0.00046 ***
location_wildlifepathway:wildlife_recording_classeswildlife           144.00     158.64   0.908      0.37061
location_wildlifegroup 2:wildlife_recording_classeswildlife_species   108.10      67.64   1.598      0.11956
location_wildlifegroup1:wildlife_recording_classeswildlife_species     47.30      67.64   0.699      0.48930
loaction_wildlifepathway:wildlife_recording_classeswildlife_species       NA         NA      NA           NA
```

Generalized Linear Model, fixed factors

Comparison effect of factors on horse group 1 and 2

Full Model

```
glm(formula = group_num ~ sex.num + breed_type_num + 
    age, family = binomial(logit), data = Dataset)

Deviance Residuals: 
     Min        1Q    Median        3Q       Max  
-1.85833  -0.00009   0.44057   0.56948   1.06501  

Coefficients:
                Estimate Std. Error z value Pr(>|z|)
(Intercept)     -23.3593  5176.1172  -0.005    0.996
sex.num           1.6532     2.1205   0.780    0.436
breed_type_num   20.9283  5176.1148   0.004    0.997
age               0.1801     0.1980   0.910    0.363

(Dispersion parameter for binomial family taken to be 1)

    Null deviance: 15.1582  on 10  degrees of freedom
Residual deviance:  7.9448  on  7  degrees of freedom
  (1 observation deleted due to missingness)
AIC: 15.945
```

Reduced model

```
glm(formula = group_num ~ sex.num, family = binomial(logit),
    data = Dataset)

Deviance Residuals:
    Min       1Q   Median       3Q      Max
-1.8930  -0.9005   0.6039   0.6039   1.4823

Coefficients:
            Estimate Std. Error z value Pr(>|z|)
(Intercept)  -0.6931     0.8660  -0.800   0.4235
sex.num       2.3026     1.3964   1.649   0.0992 .
---
Signif. codes:  0 '***' 0.001 '**' 0.01 '*' 0.05 '.' 0.1 ' ' 1

(Dispersion parameter for binomial family taken to be 1)

    Null deviance: 16.301  on 11  degrees of freedom
Residual deviance: 13.045  on 10  degrees of freedom
AIC: 17.045
```

Frequency Analysis

Frequencies wolves at daytimes

```
 Chi-squared test for given probabilities

data:  c(759, 85, 126)
X-squared = 883.14, df = 2, p-value < 2.2e-16
```

Frequencies Wolf movement types

```
Chi-squared test for given probabilities

data:  c(695, 117, 25, 3, 9)
X-squared = 2080.5, df = 4, p-value < 2.2e-16
```

Frequencies wildlife total observations

```
Chi-squared test for given probabilities

data:  c(1342, 867, 810, 95)
X-squared = 1019.3, df = 3, p-value < 2.2e-16
```

Frequencies horse behaviour

```
Chi-squared test for given probabilities

data:  c(2048, 24, 1, 55, 40)
X-squared = 7517.2, df = 4, p-value < 2.2e-16
```

Frequencies wildlife at the pasture of group 1 (01.01.22 – 23.03.22)

```
Chi-squared test for given probabilities

data:  c(396, 202, 22, 10, 3)
X-squared = 932.66, df = 4, p-value < 2.2e-16
```

Frequencies wildlife at the pasture of group 2 (01.01.22 – 23.03.22)

```
Chi-squared test for given probabilities

data:  c(796, 294, 139, 8, 2, 2)
X-squared = 2334.1, df = 5, p-value < 2.2e-16
```
